# The spacing words and pseudowords reading test: normative data to measure peripheral acquired dyslexia in the Italian adult population

**DOI:** 10.3389/fpsyg.2026.1911950

**Published:** 2026-09-03

**Authors:** Laura Veronelli, Roberta Daini, Silvia Primativo, Carlo Toneatto, Lucia Ghielmi, Silvia Pino, Giulia Gilardone, Marialuisa Martelli, Lisa S. Arduino

**Affiliations:** 1 Department of Psychology, University of Milano-Bicocca and Milan Centre for Neuroscience (NeuroMI), Milan, Italy; 2 Department of Neurorehabilitation Sciences, Casa di Cura IGEA, Milan, Italy; 3 Optics and Optometry Research Centre University of Milano Bicocca (COMiB), Milan, Italy; 4 IRCCS Fondazione Don Carlo Gnocchi ONLUS, Milan, Italy; 5 Department of Human Sciences, LUMSA University, Rome, Italy; 6 Department of Psychology, University of Milano-Bicocca, Milan, Italy; 7 Department of Psychology, University La Sapienza, Rome, Italy

**Keywords:** acquired peripheral dyslexia, crowding, neglect dyslexia, normative data, omissions, reading test, substitutions

## Abstract

**Introduction:**

Recent studies in right-brain-damaged patients with an acquired reading disorder have shown that omission and substitution errors in reading single words and pseudowords may result from two different mechanisms: the association of spatial neglect with altered eye movements (neglect dyslexia), which produces left-sided omissions, or an enhancement of a crowding phenomenon (crowding dyslexia), responsible for less lateralized substitutions. This study aims to provide normative data for the Spacing Words and Pseudowords (SWAP) reading test for the Italian population, a novel tool sensitive to identify specific visuo-perceptual and attentional deficits in patients with acquired peripheral dyslexia based on the modulatory effect of letter spacing.

**Methods:**

The test was administered to 199 neurologically unimpaired Italian participants (86 males), with an average age of 57.3 years (range 20–90) and an average education of 14.1 years (range 5–28). An innovative scoring method based on the type and position of errors (letter-based) was proposed, and the effect of spacing condition on specific types of errors was investigated.

**Results:**

Regression-based norms are provided for both word-based and letter-based errors. A significant effect of spacing on accuracy, with an increased number of errors in the spaced compared to the unspaced condition, was observed for substitutions and additions, but not for omissions. Furthermore, examples to illustrate the clinical application of the test in right-brain-damaged patients with acquired dyslexia are described.

**Discussion:**

The SWAP reading test, provided with proper norms, may serve as a clinical tool to investigate visual-perceptual and attentional mechanisms involved in reading and possibly affected by brain damage, disentangling neglect dyslexia from crowding dyslexia.

## Introduction

Reading is a complex process involving the visual analysis of a sequence of letters, access to meaning and phonology, as well as, when reading aloud, the motor planning and control of language production (see Carreiras et al., 2014, for review). In reading, it is possible to distinguish between central and peripheral stages (Coslett, 2000; Coslett and Turkeltaub, 2016). Central processes refer to the phases that involve the use of semantic and lexical knowledge. Sublexical conversion processes are also considered central, allowing the transformation of letter sequences that do not correspond to words into sequences of sounds (grapheme–phoneme conversion, used in reading aloud), essential for learning new lexical units. Peripheral processes instead concern the low-level visual-perceptual/attentional analyses imposed on word recognition, as well as working memory mechanisms and the motor-planning components required by the task (Starrfelt and Woodhead, 2021).

Acquired reading disorders are frequent after brain damage (Coslett and Turkeltaub, 2016). The broad term “acquired dyslexia” refers to reading deficits in previously literate individuals due to brain damage or neural dysfunction. Depending on whether central or peripheral processes are impaired, reading disorders are typically classified into central or peripheral dyslexias. While central dyslexias are considered linguistic disorders, peripheral dyslexias affect other cognitive functions. Consistently, in most cases, the former typically result from left-hemisphere lesions, whereas the latter are more commonly associated with right-hemisphere damages.

Among peripheral dyslexias, one of the most investigated disorders is neglect dyslexia (ND; see Moore and Demeyere, 2017; Vallar et al., 2010 for a review). Right-brain-damaged patients with spatial neglect (SN) and ND typically make errors in the left side of written words. Errors also occur when reading sentences or text, although errors in single words are considered more specific to ND (Stenneken et al., 2008).

According to the first classification proposed by Ellis et al. (1987), in left ND misread words and targets are “identical to the right of an identifiable neglect point in each word but share no letters in common to the left of the neglect point” (cit. p. 445). The authors considered misread words to reflect SN only when omissions and substitutions are limited to the left side of the neglect point (with the right side identical to the target). Additions were rarer and consisted of adding letters to the beginning of the target word so that the neglect point was before the beginning of the word itself. “Others” errors were cases in which no neglect point could be identified or in which the errors were based on the visual similarity between the target word and the misspelled word. Such a criterion implies that many of the errors produced by the patients are not considered as neglect errors in the diagnosis of ND. A similar, though more lenient criterion for substitutions is that the target and the response (i.e., the paralexic error) may share some letters occupying the left portion of the string (Caramazza and Hillis, 1990).

However, experience with patients with ND has shown that most of the time a single reading error can involve both omissions and substitutions, affecting not only the left side but even the right side of the letter string. For this reason, Martelli et al. (2011) used a more data-driven analysis to count letter errors across the entire word. Through this error analysis, the authors found that omission and substitution errors observed in right-brain-damaged ND patients, as classified using Ellis’s criterion, are differentially affected by letter spacing. As expected, increasing letter spacing—enlarging the stimulus—produced an increase in letter omissions on the left side of the space. Notably, increasing letter spacing reduces letter substitution errors both on the left as well as the right side of the word.

The authors proposed that letter-based omission and substitution errors may be due to two independent functional mechanisms: (i) a visuospatial dysfunction that would be predominantly responsible for omissions and underlies both ND and SN manifestations; and (ii) a perceptual/attentional deficit independent of SN that would determine the prevalence of substitutions. Subsequent studies on different patient cohorts demonstrated that, whereas omissions are associated with SN, substitutions depend on visual crowding (Daini et al., 2013, 2021, 2025; Primativo et al., 2013, 2015). These results are consistent with other evidence from German-speaking patients, showing that only omissions are related to ND (Weinzierl et al., 2012).

Crowding is a visual phenomenon in which the ability to identify a stimulus or a letter is compromised when it is surrounded by other nearby elements. This would especially happen in peripheral vision, where the crowding range (the center-to-center letter spacing needed to restore target recognition) is larger than in foveal vision (Andriessen and Bouma, 1976). In reading, increasing letter spacing improves recognition of words presented in peripheral vision (Bricolo et al., 2015). Interestingly, Daini et al. (2021, 2025) found that acquired reading dyslexia, characterized by substitutions, is not only sensitive to letter spacing but also dissociated from SN, and they called it crowding dyslexia (CD).

Another relevant aspect is that ND patients, like patients with CD, tend to make more reading errors on pseudowords than on words, showing the classical lexical effect (see Arduino et al., 2002). This also occurs in Italian patients, whose language is considered orthographically transparent (but see Làdavas et al., 1997). Moreover, patients have been shown to be sensitive to the lexical and morphological status of a word, written word frequency, orthographic neighborhood, and the presence of morphological constituents as suffixes or compound nouns (Arduino et al., 2002; Semenza et al., 2011), as well as to word stress (Cubelli and Beschin, 2005; Rusconi et al., 2004).

The hypothesis that SN is a disorder of spatial awareness (e.g., Bisiach et al., 1979) implies that evidence of unconscious processing should also be considered in reading. Patients with ND show better performance in implicit tasks, such as lexical and semantic decision, word and sentence bisection tasks, association tasks, and Stroop tasks (e.g., Arduino et al., 2003; Veronelli et al., 2014a,b, 2020a). This result supports the hypothesis that lexical and semantic processes are generally preserved in right-brain-damaged patients with ND (Vallar et al., 2010) due to the superiority of the left hemisphere to process language (Veronelli and Vallar, 2025; Bonandrini et al., 2020).

Although several tools are available in Italian for the assessment of word processing in central dyslexias (e.g., the word and pseudoword reading test of the *Batteria per l’Analisi dei Deficit Afasici*, BADA, by Miceli et al., 1994; the reading subtest of the *Battery for the Assessment of Severe Acquired Lexical Damage in Italian*; by Veronelli et al., 2020b), to date, no diagnostic tests have been developed to assist clinicians in assessing acquired peripheral dyslexia and differentiating error patterns consistent with ND *versus* CD.

In experimental studies, lists of words and pseudowords in two different spatial conditions (letters spaced and unspaced) have proven to be an effective and sensitive paradigm in the identification of ND and CD in right-brain-damaged patients’ reading behavior (Daini et al., 2013, 2021; Martelli et al., 2011; Primativo et al., 2015). In particular, ND patients would show left-side omissions, increasing with letter spacing. On the other hand, CD patients would show less lateralized substitutions decreasing with spacing, unrelated to SN.

Here, we described the Spacing Words and Pseudowords (SWAP) reading test, a reading aloud test designed to assess acquired peripheral dyslexias, which includes a wide subset of stimuli used in the studies cited above (Daini et al., 2021, 2025; Primativo et al., 2015), namely words and pseudowords (i.e., legal nonwords), presented in both spaced and unspaced formats. This test allows the understanding of how the reading errors’ typology may be differently affected by letter spacing, reflecting deficits of different mechanisms.

The study reports on normative data with neurologically unimpaired Italian participants, stratified by age, sex, and education level, in order to measure and classify the types of reading errors. Establishing normative data is essential to determine whether a patient’s performance significantly deviates from reference values from a healthy population. We expect that these results will refine diagnostic testing and subsequent treatment.

The results will provide neuropsychologists with norms for a clinical tool useful in the evaluation of prelexical components involved in reading, such as crowding, not addressed in the available clinical tests.

Furthermore, a new method of evaluating reading errors is proposed, not limited to the classic *word-based analysis* (total number of incorrectly read words), but extending to a *letter-based analysis*, allowing a more refined evaluation of the types of errors produced (i.e., omissions, substitutions, and additions).

Finally, examples to illustrate the clinical application of the test in right-brain-damaged patients with acquired dyslexia are provided.

## Materials and methods

### Participants

For the research project, a sample of 200 Italian neurologically unimpaired volunteer participants was recruited through direct contact. Participants underwent a clinical interview to exclude the presence of known developmental dyslexia, uncorrected visual disturbances, and previous psychiatric and neurological problems. As a confirmation of normal or corrected-to-normal vision, each participant was presented with a 0.1 logMAR measured with the Milan Eye Charts (MEC; Facchin et al., 2019), placed at 3 m. A 24-year-old, graduate male produced a much higher number of errors compared to participants with the same age and education level (9 errors in reading list A and 12 errors in reading list B pseudowords), and he was excluded from the sample because of the possible presence of undiagnosed developmental dyslexia.

A final sample of 199 participants (86 males), aged 20–90 years, was recruited, with a mean age of 57.3 years (SD = 21.0; range 20–90) and mean education of 14.1 years (SD = 4.43; range 5–28). Participants aged 65 years or older scored within the normal range on the Montreal Cognitive Assessment (Aiello et al., 2022). This sample size is comparable to those adopted in previous regression-based normative studies. Table 1 describes the different groups of participants, classified according to age and years of education.

**Table 1:** Sample distribution (males + females).

Age	Education						<8	8–12	13–16	≥17	Total
20–40	–	3 (2 + 1)	21 (9 + 12)	20 (8 + 12)	44 (19 + 25)
41–50	–	7 (6 + 1)	6 (1 + 5)	8 (1 + 7)	21 (8 + 13)
51–60	–	5 (4 + 1)	14 (4 + 10)	13 (7 + 6)	32 (15 + 17)
61–70	–	9 (3 + 6)	12 (6 + 6)	13 (5 + 8)	34 (14 + 20)
71–80	8 (3 + 5)	14 (4 + 10)	8 (4 + 4)	7 (1 + 6)	37 (12 + 25)
81–90	7 (2 + 5)	9 (6 + 3)	8 (6 + 2)	7 (4 + 3)	31 (18 + 13)
Total	15 (5 + 10)	47 (25 + 22)	69 (30 + 39)	68 (26 + 42)	199 (86 + 113)

Groups of participants classified according to age indicated in rows and years of education in columns.

### Stimuli

The word stimuli consisted of 72 singular Italian nouns, taken from the dataset of Barca et al. (2002). Stimuli are morphologically simple nouns, that is, not derived and not compound, not ambiguous with regard to grammatical category and meaning, and have a length of five or seven letters, without double consonants and with a 1:1 letter-to-sound correspondence, except for the phonemes “sc” (included in five items) and “gn” (one item). A total of 72 pseudowords, that is, legal nonwords, were generated from words in which at least two letters were substituted in random positions while preserving pronunciation and minimizing similarity between words.

The final 144 stimuli (72 words and 72 pseudowords) were divided into list A and list B. Each list contained 72 stimuli, balanced for length (half 5-letter and half 7-letter) and distributed across four sub-lists of 18 items each: (i) unspaced words, (ii) unspaced pseudowords, (iii) spaced words, and (iv) spaced pseudowords.

**Figure 1:**
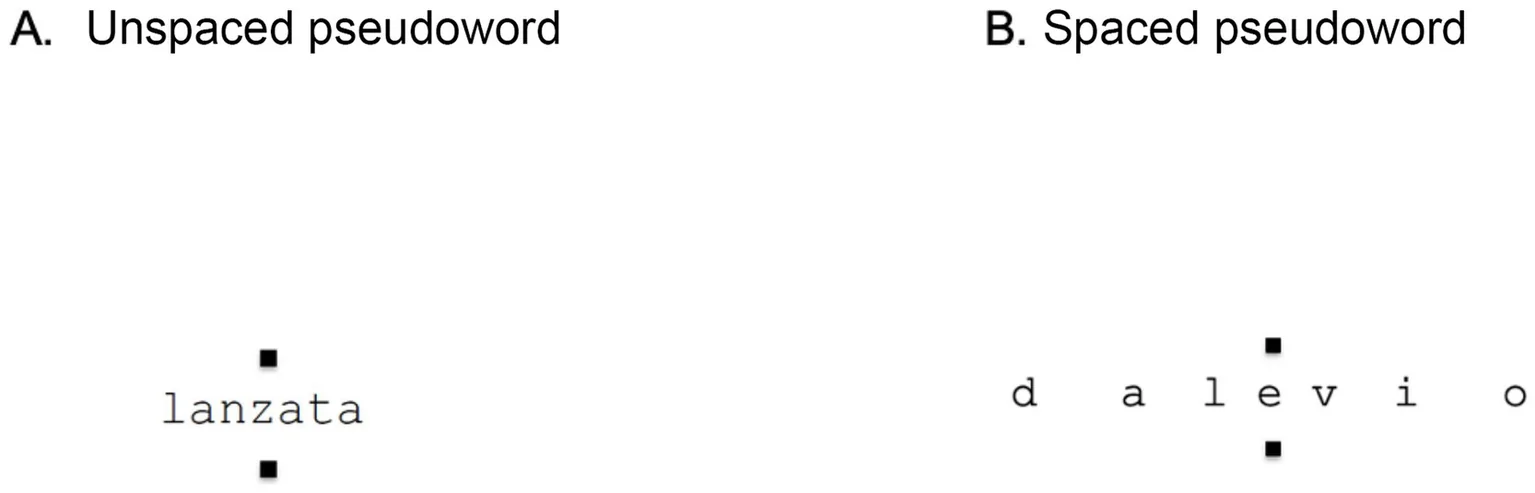
Examples of unspaced (Panel A) and spaced (Panel B) stimuli included in the test.

Unlike the “unspaced” condition, in which letters were presented with standard spacing, the “spaced” condition incorporates gradual spacing between letters, with a distance between them increasing by a factor of 2 as a function of letter eccentricity (Figures 1A,B). The “crowding effect” increases as the visual eccentricity increases: the greater the eccentricity, that is, the greater the distance of the stimulus from the center of the visual field, the greater the “crowding effect.” According to Bouma’s law (Bouma, 1970), the crowding range increases with a proportionality of about 0.5 the target viewing eccentricity (i.e., if a letter is located at 4° of eccentricity the center-to-center spacing between the target and the surrounding letters needs to be greater than or equal to 2° to restore recognition).

Stimuli (both words and pseudowords) were matched for specific psycholinguistic variables. For words, the variables considered were age of acquisition (AoA), familiarity (FAM), imageability (IMM), concreteness (CONC), written word frequency (FQ), number of letters (LETTERS), number of syllables (SYLL), orthographic neighborhood size (N-SIZE), and bigram frequency (FQ-BIGR). For pseudowords, we considered number of letters (LETTERS), number of syllables (SYLL), orthographic neighborhood size (N-SIZE), and bigram frequency (FQ-BIGR). The orthographic neighbors of words correspond to the number of words that differ by just one letter from the target word, maintaining the identity and position of the other letters (for example, the word “fango” has as orthographic neighbors the words “tango” and “mango”). The frequency of bigrams instead concerns the frequency with which adjacent pairs of letters appear in printed texts (for example, in the Italian language, the bigram “qu” is common, while “qz” is rare) (see Barca et al., 2002, for a detailed description of the lexical and sublexical variables taken into consideration).

It is worth mentioning that the matching of psycholinguistic variables was made between words within the same list (e.g., word unspaced vs. word spaced in list A; the same for list B) and between lists (word unspaced in list A vs. word unspaced in list B; the same for spaced words). This matching allowed us to explore the effects of the two variables taken into consideration, namely lexicality and spacing, net of some psycholinguistic variables. Mean values of the psycholinguistic variables for words and pseudowords and the list of stimuli included in the battery are reported as Supplementary materials.

### Procedure

The presentation order of list A and B was counterbalanced across participants. For each list, the fixed presentation order of the sub-lists was: unspaced words, unspaced pseudowords, spaced words, spaced pseudowords.

Stimuli were written in black, lowercase print, Courier New, 22 pt., and were placed in the center of a white screen, between two fixation points positioned centrally, one above and one below the word to be read (see Figure 1). The reading test was conducted on a 13-inch screen computer, in person and individually, at a distance of 57 cm from the screen.

During the testing session, participants were asked to read aloud the stimuli without time pressure. Each sub-list was separated by a white screen that warned the subject about the type of stimuli to read (i.e., words or pseudowords, spaced and unspaced). The participants’ performance was audio-recorded in order to allow remote assessment of response accuracy. The first self-correction was accepted as a correct response.

Two correction criteria were used: word-based and letter-based errors. The word-based criterion (WBC) referred to the total number of errors made on the stimulus (range 0–72 for each list; range 0–18 for each sub-list), while the letter-based criterion (LBC) considered the type of error committed on each letter of the stimulus, distinguishing omissions, substitutions (range 0–432 for each list; range 0–108 for each sub-list), and additions (Martelli et al., 2011). This type of correction is particularly important in languages such as Italian, with a transparent orthography (1:1 letter-to-sound correspondence).

It is worth mentioning that, according to the LBC, it was possible for more than one letter-based error to be calculated on the same pseudoword (e.g., “rofando” read as “ofondo” would lead to the count of one substitution and one omission). As addition errors cannot be mapped to a specific target letter, it was not possible to calculate an error range or establish a maximum number of errors for this category.

Although the number of errors can be manually calculated, a computerized, automated correction procedure, which permits counting errors according to the WBC and the LBC, and classifying them as omissions and substitutions, is made available (see the OSF link below). The complete version of the test is available upon request from the authors.

### Statistical analyses

A normative sample of neurologically healthy participants, stratified by sex, age, and education level, was collected in order to examine the type of errors made in reading lists of words and pseudowords, both unspaced and spaced. As often occurs in reading tasks, a ceiling effect was observed in the present study: no participant made errors in reading real words, both unspaced and spaced, and no further analyses were performed for this type of stimulus. Conversely, in pseudoword reading, some errors were observed also in the neurologically unimpaired population. Errors in pseudoword reading were analyzed, and cutoff scores were calculated. First, we verified the need to adjust scores according to demographic variables (sex, age, and years of education), estimating regression-based models for each covariate. Due to the evidence of overdispersion, negative binomial regressions were applied for each continuous covariate (age and years of education, see Veronelli et al., 2025a, for a similar approach). For LBC-based omission and addition errors in reading unspaced stimuli of both lists and omission errors in spaced stimuli of list B, Poisson regression models were used, as the deviance-to-degrees-of-freedom ratio indicated no overdispersion. In both cases, predictors were included in a stepwise regression together with sex (dummy-coded) in order to establish the significant predictors to condition the adjusted scores. Predictors were entered in their original metric as continuous variables, as quadratic terms did not improve model fit. Adjustment formulas were derived from the most parsimonious regression model retained for each variable. It should be noted that in models with a log link, predictor effects are estimated on the logarithmic scale: the adjustment formula requires dividing the exponential of the weighted covariate scores rather than subtracting them to obtain an adjusted score in the original outcome scale.

Then, outer and inner nonparametric tolerance limits (oTL and iTL, respectively) were computed. As higher scores indicate a greater number of errors, cutoff scores were established for performances exceeding the one-sided nonparametric 95% tolerance limit at the 95% confidence level (the 5th observation for 199 participants; Aiello and Depaoli, 2022). A borderline performance comprised scores between the oTL and iTL, and performance below the iTL was classified as within the normal range.

Furthermore, Spearman’s correlations were run for WBC errors in unspaced pseudowords of list A and B and spaced pseudowords of list A and B in order to verify the possibility of using the two lists as parallel forms.

To evaluate the effect of spacing on reading errors according to the WBC, given the non-normal distribution of errors, Wilcoxon signed-rank tests were used. Errors of unspaced and spaced pseudowords in the two lists, A and B, were analyzed separately.

For the LBC analysis, since the number of letter-based errors was very limited for the three types of errors considered (omissions, substitutions, and additions), errors for the two lists were merged (list A + list B), keeping the spacing variable separate. In this way, six variables were obtained: unspaced omissions, unspaced substitutions, unspaced additions, spaced omissions, spaced substitutions and spaced additions. To investigate the effect of the error type in relation to spacing, separate non-parametric Friedman tests were conducted with post-hoc Durbin–Conover pairwise comparisons.

Statistical analyses were conducted using Jamovi (The jamovi project, 2025, version 2.6). This research project has been evaluated by the Research Evaluation Committee of the Department of Psychology of the University of Milano-Bicocca for minimal-risk studies (Protocol No. RM-2023-738) and by the CET Lombardia 3 (Protocol No. 4924_SA_05.11.2025_P_bis). Written informed consent was obtained from all participants before testing.

## Results

As already reported, no errors were committed by neurologically unimpaired participants in reading unspaced and spaced words, and no statistical analyses were conducted for these stimuli. Tables 2, 3 summarize the mean number of errors in reading pseudowords using the WBC and the LBC, divided by type of error, namely omissions, substitutions, and additions, made by the entire group of participants in list A and B, respectively. Participants showed more substitutions than omissions and additions.

**Table 2:** Mean number (SD) of word-based (WBC) and letter-based errors (LBC) made in list A pseudoword reading, stratified by participants’ age and education ranges.

Age	Education	WBC unspaced	WBC spaced	LBC-omission unspaced	LBC-substitution unspaced	LBC-addition unspaced	LBC-omission spaced	LBC-substitution spaced	LBC-addition spaced
20–40	<8	–	–	–	–	–	–	–	–
	8–12	0.33 (0.58)	0.00 (0.00)	0.00 (0.00)	0.00 (0.00)	0.33 (0.58)	0.00 (0.00)	0.00 (0.00)	0.00 (0.00)
	13–16	0.43 (0.75)	0.67 (0.66)	0.05 (0.22)	0.24 (0.54)	0.14 (0.36)	0.10 (0.44)	0.14 (0.36)	0.43 (0.60)
	≥17	0.45 (0.69)	0.40 (0.68)	0.00 (0.00)	0.35 (0.67)	0.10 (0.31)	0.00 (0.00)	0.20 (0.41)	0.20 (0.41)
41–50	<8	–	–	–	–	–	–	–	–
	8–12	0.57 (0.53)	0.57 (1.51)	0.00 (0.00)	0.57 (0.53)	0.00 (0.00)	0.00 (0.00)	0.29 (0.76)	0.29 (0.76)
	13–16	0.17 (0.41)	0.50 (1.22)	0.00 (0.00)	0.17 (0.41)	0.00 (0.00)	0.00 (0.00)	0.50 (1.22)	0.00 (0.00)
	≥17	0.63 (0.74)	0.75 (0.89)	0.13 (0.35)	0.50 (0.76)	0.00 (0.00)	0.13 (0.35)	0.50 (0.76)	0.13 (0.35)
51–60	<8	–	–	–	–	–	–	–	–
	8–12	0.40 (0.55)	0.80 (1.30)	0.00 (0.00)	0.40 (0.55)	0.00 (0.00)	0.00 (0.00)	0.80 (1.30)	0.00 (0.00)
	13–16	0.50 (0.76)	0.50 (0.85)	0.00 (0.00)	0.43 (0.76)	0.07 (0.27)	0.00 (0.00)	0.36 (0.74)	0.14 (0.36)
	≥17	0.15 (0.38)	0.85 (1.52)	0.00 (0.00)	0.15 (0.38)	0.00 (0.00)	0.15 (0.38)	0.54 (1.33)	0.15 (0.38)
61–70	<8	–	–	–	–	–	–	–	–
	8–12	0.13 (0.35)	1.00 (1.07)	0.00 (0.00)	0.13 (0.35)	0.00 (0.00)	0.00 (0.00)	0.63 (0.92)	0.38 (0.52)
	13–16	0.23 (0.60)	0.85 (1.14)	0.08 (0.28)	0.08 (0.28)	0.08 (0.28)	0.31 (0.85)	0.38 (0.51)	0.15 (0.38)
	≥17	0.23 (0.44)	0.15 (0.38)	0.00 (0.00)	0.23 (0.44)	0.00 (0.00)	0.00 (0.00)	0.08 (0.28)	0.08 (0.28)
71–80	<8	1.50 (1.51)	1.75 (1.28)	0.13 (0.35)	1.00 (1.31)	0.38 (0.52)	0.25 (0.46)	0.88 (0.64)	0.63 (0.92)
	8–12	0.21 (0.58)	0.43 (0.65)	0.00 (0.00)	0.21 (0.58)	0.00 (0.00)	0.00 (0.00)	0.36 (0.63)	0.07 (0.27)
	13–16	0.25 (0.71)	0.75 (1.16)	0.00 (0.00)	0.25 (0.71)	0.00 (0.00)	0.13 (0.35)	0.50 (1.07)	0.13 (0.35)
	≥17	0.14 (0.38)	0.86 (1.86)	0.00 (0.00)	0.14 (0.38)	0.00 (0.00)	0.14 (0.38)	0.43 (0.13)	0.29 (0.49)
81–90	<8	1.00 (1.00)	1.29 (1.11)	0.00 (0.00)	0.86 (1.07)	0.14 (0.38)	0.14 (0.38)	0.29 (0.49)	0.86 (1.07)
	8–12	0.44 (0.73)	1.44 (1.74)	0.11 (0.33)	0.33 (0.71)	0.00 (0.00)	0.00 (0.00)	1.00 (1.32)	0.44 (0.53)
	13–16	0.25 (0.46)	1.25 (0.46)	0.00 (0.00)	0.25 (0.46)	0.00 (0.00)	0.00 (0.00)	0.25 (0.46)	0.00 (0.00)
	≥17	0.43 (0.79)	0.14 (0.38)	0.00 (0.00)	0.14 (0.38)	0.29 (0.49)	0.00 (0.00)	0.14 (0.38)	0.00 (0.00)

**Table 3:** Mean number (SD) of word-based (WBC) and letter-based errors (LBC) made in list B pseudoword reading, stratified by participants’ age and education ranges.

Age	Education	WBC unspaced	WBC spaced	LBC-omission unspaced	LBC-substitution unspaced	LBC-addition unspaced	LBC-omission spaced	LBC-substitution spaced	LBC-addition spaced
20–40	<8	–	–	–	–	–	–	–	–
	8–12	0.33 (0.58)	0.33 (0.58)	0.00 (0.00)	0.00 (0.00)	0.33 (0.58)	0.00 (0.00)	0.33 (0.58)	0.00 (0.00)
	13–16	0.19 (0.40)	0.81 (0.93)	0.05 (0.22)	0.14 (0.36)	0.00 (0.00)	0.00 (0.00)	0.52 (0.75)	0.29 (0.56)
	≥17	0.10 (0.45)	0.65 (0.99)	0.00 (0.00)	0.10 (0.45)	0.00 (0.00)	0.05 (0.22)	0.35 (0.75)	0.25 (0.72)
41–50	<8	–	–	–	–	–	–	–	–
	8–12	1.43 (1.27)	0.86 (1.07)	0.00 (0.00)	1.14 (1.07)	0.29 (0.49)	0.14 (0.38)	0.71 (0.76)	0.00 (0.00)
	13–16	0.00 (0.00)	0.33 (0.52)	0.00 (0.00)	0.00 (0.00)	0.00 (0.00)	0.00 (0.00)	0.17 (0.41)	0.17 (0.41)
	≥17	0.13 (0.35)	0.75 (1.49)	0.00 (0.00)	0.13 (0.35)	0.00 (0.00)	0.13 (0.35)	0.25 (0.71)	0.38 (0.74)
51–60	<8	–	–	–	–	–	–	–	–
	8–12	0.60 (0.89)	0.80 (0.45)	0.00 (0.00)	0.40 (0.55)	0.20 (0.45)	0.00 (0.00)	0.00 (0.00)	0.80 (0.45)
	13–16	0.36 (0.63)	1.00 (1.18)	0.00 (0.00)	0.36 (0.63)	0.00 (0.00)	0.00 (0.00)	0.43 (0.94)	0.57 (0.85)
	≥17	0.08 (0.28)	0.69 (1.03)	0.00 (0.00)	0.08 (0.28)	0.00 (0.00)	0.00 (0.00)	0.46 (0.88)	0.23 (0.44)
61–70	<8	–	–	–	–	–	–	–	–
	8–12	0.75 (0.89)	0.75 (0.89)	0.25 (0.46)	0.25 (0.46)	0.25 (0.46)	0.00 (0.00)	0.75 (0.89)	0.00 (0.00)
	13–16	0.92 (1.12)	1.23 (1.01)	0.00 (0.00)	0.77 (0.93)	0.15 (0.38)	0.00 (0.00)	1.00 (0.91)	0.23 (0.44)
	≥17	0.08 (0.28)	0.31 (0.63)	0.00 (0.00)	0.00 (0.00)	0.08 (0.28)	0.00 (0.00)	0.15 (0.55)	0.15 (0.38)
71–80	<8	0.50 (0.53)	1.25 (1.39)	0.13 (0.35)	0.38 (0.52)	0.00 (0.00)	0.38 (0.52)	0.63 (1.06)	0.25 (0.46)
	8–12	0.36 (0.84)	0.57 (0.94)	0.00 (0.00)	0.36 (0.84)	0.00 (0.00)	0.00 (0.00)	0.43 (0.85)	0.14 (0.36)
	13–16	0.63 (1.06)	2.25 (1.67)	0.00 (0.00)	0.63 (1.06)	0.00 (0.00)	0.00 (0.00)	1.88 (1.36)	0.38 (0.74)
	≥17	0.57 (1.51)	0.57 (1.13)	0.00 (0.00)	0.57 (1.51)	0.00 (0.00)	0.00 (0.00)	0.57 (1.13)	0.00 (0.00)
81–90	<8	0.86 (0.90)	1.43 (1.13)	0.00 (0.00)	0.57 (0.79)	0.29 (0.49)	0.14 (0.38)	0.71 (0.76)	0.57 (0.98)
	8–12	0.78 (1.30)	1.00 (1.50)	0.00 (0.00)	0.56 (0.88)	0.22 (0.44)	0.00 (0.00)	0.89 (1.54)	0.11 (0.33)
	13–16	0.63 (0.74)	1.00 (1.41)	0.00 (0.00)	0.63 (0.74)	0.00 (0.00)	0.25 (0.46)	0.50 (0.76)	0.25 (0.46)
	≥17	0.71 (0.95)	1.29 (1.11)	0.00 (0.00)	0.71 (0.95)	0.00 (0.00)	0.00 (0.00)	1.29 (1.11)	0.00 (0.00)

### List A

#### WBC unspaced pseudowords

For WBC unspaced pseudowords, bivariate negative binomial regressions indicated education as a significant predictor (*β* = −0.064, *z* = −2.26, *p* = 0.024), while age was not (*β* = −0.000, *z* = 0.081, *p* = 0.935). Stepwise negative binomial regression with education and sex indicated that sex was not a significant predictor (*β* = −0.002, *z* = −0.010, *p* = 0.992). The final model included only education, and the adjustment formula is:

$$\text{Yadj}=\text{Yraw}/\exp[-0.064^{*}(\text{education}-14.06)]$$

#### WBC-spaced pseudowords

For WBC-spaced pseudowords, results indicated education as a significant predictor (*β* = −0.056, *z* = −2.21, *p* = 0.027), while age was not (*β* = 0.009, *z* = 1.67, *p* = 0.096). Stepwise negative binomial regression with education and sex indicated that sex was not a significant predictor (*β* = −0.424, *z* = −1.92, *p* = 0.055). The final model included only education, and the adjustment formula is:

$$\text{Yadj}=\text{Yraw}/\exp[-0.056^{*}(\text{education}-14.06)]$$

#### LBC unspaced pseudowords

##### Omissions

For omission errors in unspaced pseudowords, no significant predictors were found (age: *β* = 0.002, *z* = 0.0929, *p* = 0.926; education: *β* = −0.00757, *z* = −0.742, *p* = 0.458; Sex: *β* = 0.1701, *z* = 0.186, *p* = 0.852), and no adjustment to the raw score is needed.

##### Substitutions

For substitution errors in unspaced pseudowords, bivariate negative binomial regressions indicated education as a significant predictor (*β* = −0.0646, *z* = −1.98, *p* = 0.048), while age was not (*β* = 0.0027, *z* = 0.387, *p* = 0.699). Stepwise negative binomial regression with education and sex indicated that sex was not a significant predictor (*β* = 0.0374, *z* = 0.129, *p* = 0.897). The final model included only education, and the adjustment formula is:

$$\text{Yadj}=\text{Yraw}/\exp[-0.0646^{*}(\text{education}-14.06)]$$

##### Additions

For addition errors in unspaced pseudowords, no significant predictors were found (age: *β* = −0.009, *z* = −0.772, *p* = 0.440; education: *β* = −0.0623, *z* = −1.02, *p* = 0.306; sex: *β* = −0.2408, *z* = −0.45, *p* = 0.653) and no adjustment to the raw score is needed.

#### LBC spaced pseudowords

##### Omissions

For omission errors in spaced pseudowords, no significant predictors were found (age: *β* = 0.008, *z* = 0.510, *p* = 0.610; education: *β* = 0.0015, *z* = 0.0197, *p* = 0.984; sex: *β* = −0.8663, *z* = −1.305, *p* = 0.192), and no adjustment to the raw score is needed.

##### Substitutions

For substitution errors in spaced pseudowords, bivariate negative binomial regressions indicated age as a significant predictor (*β* = 0.0170, *z* = 2.29, *p* = 0.022), while education was not (*β* = −0.0478, *z* = −1.45, *p* = 0.148). Stepwise negative binomial regression with age and sex indicated that both variables were significant predictors and, they were included in the final model (age: *β* = 0.0172, *z* = 2.35, *p* = 0.019; sex: *β* = −0.7093, *z* = −2.47, *p* = 0.014). For the calculation of adjusted scores, sex was recoded −0.5 for males and +0.5 for females. Accordingly, the adjustment formulas are:

$$\text{For females}:\text{Yadj}=\text{Yraw}/\exp[(+0.0172^{*}(\mathrm{age}-57.33))-0.3546]$$

$$\text{For males}:\text{Yadj}=\text{Yraw}/\exp[(+0.0172^{*}(\mathrm{age}-57.33))+0.3546]$$

##### Additions

For addition errors in spaced pseudowords, bivariate negative binomial regressions indicated education as a significant predictor (*β* = −0.104, *z* = −3.00, *p* = 0.003), while age was not (*β* = −0.000, *z* = −0.0521, *p* = 0.958). Stepwise negative binomial regression with education and sex indicated that sex was not a significant predictor (*β* = 0.269, *z* = 0.87, *p* = 0.384). The final model included only education, and the adjustment formula is:

$$\text{Yadj}=\text{Yraw}/\exp[-0.104^{*}(\text{education}-14.06)]$$

### List B

#### WBC unspaced pseudowords

For WBC unspaced pseudowords, bivariate negative binomial regressions indicated age (*β* = 0.0215, *z* = 3.02, *p* = 0.003) and education (*β* = −0.116, *z* = − 3.73, *p* < 0.001) to be significant predictors. Stepwise negative binomial regression with age, education, and sex indicated that only education was a significant predictor (*β* = −0.0918, *z* = −2.69, *p* = 0.007), while age (*β* = 0.0134, *z* = 1.74, *p* = 0.082) and sex (*β* = −0.439, *z* = −1.65, *p* = 0.098) were not. The final model included only education, and the adjustment formula is:

$$\text{Yadj}=\text{Yraw}/\exp[-0.116^{*}(\text{education}-14.06)]$$

#### WBC spaced pseudowords

For WBC spaced pseudowords, no significant predictors were found (age: *β* = 0.0075, *z* = 1.66, *p* = 0.097; education: *β* = −0.0382, *z* = −1.81, *p* = 0.071; sex: *β* = −0.204, *z* = −1.09; *p* = 0.276), and no adjustment to the raw score is needed.

#### LBC unspaced pseudowords

##### Omissions

For omission errors in unspaced pseudowords, no significant predictors were found (age: *β* = 0.005, *z* = 0.207, *p* = 0.836; education: *β* = −0.236, *z* = −1.86, *p* = 0.063; sex: *β* = 0.826, *z* = 0.715, *p* = 0.475), and no adjustment to the raw score is needed.

##### Substitutions

For substitution errors in unspaced pseudowords, results indicated age (*β* = 0.0229, *z* = 2.97, *p* = 0.003) and education (*β* = −0.0908, *z* = −2.72, *p* = 0.006) to be significant predictors. Stepwise negative binomial regression with age, education, and sex indicated that education and sex were not significant predictors (*β* = −0.0572, *z* = −1.61, *p* = 0.106, and *β* = −0.5427, *z* = −1.91, *p* = 0.057, respectively). The final model included only age, and the adjustment formula is:

Yadj=Yraw/exp[+0.0229∗(age−57.33)]

##### Additions

For addition errors in unspaced pseudowords, education was found to be a significant predictor (*β* = −0.210, *z* = −3.08, *p* = 0.002), while age was not (*β* = 0.018, *z* = 1.21, *p* = 0.225). Stepwise regression with education and sex indicated that only education was a significant predictor (*β* = −0.2109, *z* = −3.075, *p* = 0.002), while sex was not (*β* = −0.0832, *z* = −0.149, *p* = 0.881). The final model included only education, and the adjustment formula is:

$$\text{Yadj}=\text{Yraw}/\exp[-0.21^{*}(\text{education}-14.06)]$$

#### LBC spaced pseudowords

##### Omissions

For omission errors in spaced pseudowords, education was found as a significant predictor (*β* = −0.206, *z* = −2.52, *p* = 0.012), while age was not (*β* = 0.022, *z* = 1.19, *p* = 0.235). Stepwise regression with education and sex indicated that only education was a significant predictor (*β* = −0.2064, *z* = −2.5213, *p* = 0.012), while sex was not (*β* = −0.0127, *z* = −0.019, *p* = 0.985). The adjustment formula is as follows:

$$\text{Yadj}=\text{Yraw}/\exp[-0.206^{*}(\text{education}-14.06)]$$

##### Substitutions

For substitution errors in spaced pseudowords, bivariate negative binomial regressions indicated age as a significant predictor (*β* = 0.0130, *z* = 2.21, *p* = 0.027), while education was not (*β* = −0.0381, *z* = −1.42, *p* = 0.157). Stepwise negative binomial regression with age and sex indicated that sex was not a significant predictor (*β* = −0.4537, *z* = −1.93, *p* = 0.054). Accordingly, the adjustment formula is:

Yadj=Yraw/exp[+0.013∗(age−57.33)]

##### Additions

For addition errors in spaced pseudowords, no significant predictors were found (age: *β* = −0.0064, *z* = −0.868, *p* = 0.385; education: *β* = −0.011, *z* = −0.305, *p* = 0.760; sex: *β* = 0.359, *z* = 1.09, *p* = 0.276), and no adjustment to the raw score is needed.

The formulas to correct each score and inner and outer tolerance limits (iTL and oTL, respectively) of the SWAP reading test are reported in Tables 4, 5. A performance exceeding the cutoff value (oTL) is classified as pathological, a performance between the oTL and iTL as borderline, and a performance below the iTL as within the normal range.

**Table 4:** Age- and education-based formulas for list A and B.

List A
WBC unspaced pseudowords
Adjusted score = Raw score/exp.[−0.064 ∗ (education − 14.06)]
WBC spaced pseudowords
Adjusted score = Raw score/exp.[−0.056 ∗ (education − 14.06)]
LBC unspaced pseudowords—Omissions
No adjustment
LBC unspaced pseudowords—Substitutions
Adjusted score = Raw score/exp.[−0.0646 ∗ (education − 14.06)]
LBC unspaced pseudowords—Additions
No adjustment
LBC spaced pseudowords—Omissions
No adjustment
LBC spaced pseudowords—Substitutions
For females: Adjusted score = Raw score/exp.[(+0.0172 ∗ (age − 57.33)) − 0.3546] For males: Adjusted score = Raw score/exp.[(+0.0172 ∗ (age − 57.33)) + 0.3546]
LBC spaced pseudowords—Additions
Adjusted score = Raw score/exp.[−0.104 ∗ (education − 14.06)]

List B
WBC unspaced pseudowords
Adjusted score = Raw score/exp.[−0.116 ∗ (education − 14.06)]
WBC spaced pseudowords
No adjustment
LBC unspaced pseudowords—Omissions
No adjustment
LBC unspaced pseudowords—Substitutions
Adjusted score = Raw score/exp.[+0.0229 ∗ (age − 57.33)]
LBC unspaced pseudowords—Additions
Adjusted score = Raw score/exp.[−0.21 ∗ (education − 14.06)]
LBC spaced pseudowords—Omissions
Adjusted score = Raw score/exp.[−0.206 ∗ (education − 14.06)]
LBC spaced pseudowords—Substitutions
Adjusted score = Raw score/exp.[+0.013 ∗ (age − 57.33)]
LBC spaced pseudowords—Additions
No adjustment

**Table 5:** Cut-off and outer and inner tolerance limits (oTL and iTL, respectively) for the WBC and LBC of list A and B of the SWAP.

List A	oTL	iTL	Pathological	Borderline	Within normal range
WBC unspaced	2.41	1.68	>2.41	2.41–1.69	≤1.68
WBC spaced	3.77	2.23	>3.77	3.77–2.24	≤2.23
LBC-omission unspaced	1	0	>1	1	0
LBC-substitution unspaced	1.90	1.38	>1.90	1.90–1.39	≤1.38
LBC-addition unspaced	1	0	>1	1	0
LBC-omission spaced	1	0	>1	1	0
LBC-substitution spaced	2.71	1.89	>2.71	2.71–1.90	≤1.89
LBC-addition spaced	1.51	1.22	>1.51	1.51–1.23	≤1.22

List B	oTL	iTL	Pathological	Borderline	Within normal range
WBC unspaced	2.81	1.77	>2.81	2.81–1.78	≤1.77
WBC spaced	4	3	>4	4	≤3
LBC-omission unspaced	0	0	>0	0	0
LBC-substitution unspaced	2.30	1.33	>2.30	2.30–1.34	≤1.33
LBC-addition unspaced	0.53	0	>0.53	0.53–0	0
LBC-omission spaced	0.29	0	>0.29	0.29–0	0
LBC-substitution spaced	3.13	2.38	>3.13	3.13–2.39	≤2.38
LBC-addition spaced	2	1	>2	2	1

A performance exceeding the cut-off value (oTL) is classified as pathological, a performance between the oTL and iTL as borderline, and a performance below the iTL as within the normal range.

Test stimuli, data, as well as the computerized correction procedure (which permits counting errors according to the WBC and the LBC, and classifying them as omissions and substitutions), and the automated calculation sheet to obtain adjusted scores and classify a performance as pathological, borderline, or within the normal range are freely available at the following link: https://osf.io/vjs7y/?view_only=7430f800707c449c977bbd30110732da.

Spearman’s rank order correlations were run to determine the relationships between WBC errors in list A and B unspaced and spaced pseudowords. There was a significant positive relationship between spaced stimuli of the two lists (*r*_s_(197) = 0.187, *p* = 0.008), while between unspaced stimuli, the correlation was not significant (r_s_(197) = 0.073, *p* = 0.306).

The Wilcoxon signed-rank test, performed to evaluate the effect of spacing on reading errors according to the WBC, was significant for list A (W (198) = 1,801, *p* = 0.002, *r* = −0.34) and list B (W (198) = 1,361, *p* < 0.001, *r* = −0.52), showing an increase of errors with spacing in reading pseudowords.

To explore the effect of error type on spacing, a Friedman repeated-measures ANOVA was conducted with the three error types merged for the two lists and spacing conditions kept separate. The ANOVA revealed a significant difference between conditions (χ^2^(5) = 216, *p* < 0.001). Conover post-hoc comparisons indicated that substitutions in the spaced condition increased significantly from those in the unspaced condition (MD = 0; *M* = 0.965; MD = 0; *M* = 0.663, respectively, *p* = 0.003), as did additions (MD = 0; *M* = 0.472; MD = 0; *M* = 0.136, respectively, *p* < 0.001). However, no significant differences emerged between omissions in the spaced and unspaced conditions (MD = 0; *M* = 0.116, and MD = 0; *M* = 0.045, respectively, *p* = 0.217).

## Diagnosis of peripheral dyslexia

In the assessment of patients with right-hemisphere lesions, the SWAP reading test can be used to reveal specific patterns of acquired peripheral dyslexia. As already demonstrated in previous group studies with right-brain-damaged patients, the presence of contralesionally lateralized omissions with unspaced stimuli may indicate ND. In such cases, when spaced stimuli are administered, the number of errors is expected to increase with increasing inter-letter spacing (Daini et al., 2021, 2025).

When substitution errors are observed with unspaced stimuli—typically showing a less lateralized error distribution than that observed for omissions—a diagnosis of CD may be considered. In this condition, reading errors are expected to decrease with increased spacing, particularly with pseudowords, which are not facilitated by lexicality.

It should also be noted that in elderly participants, substitution errors may also become more frequent and increase with spacing, possibly due to a loss of the global word form representation.

### Some examples illustrating the application of SWAP reading test

To illustrate the practical application of the SWAP test, three patients with right-brain damage showing an acquired reading disorder are described and discussed individually (Table 6).

**Table 6:** Demographic, neurological, neuropsychological and SWAP reading test example data for three RBD patients.

Pt	Sex	Age	Education	Handedness	Disease duration (months)	Bell cancellation L–R omission difference	Apple test complete score	Apple test incomplete score	Omissions				Substitutions			
									Unspaced W	Spaced W	Unspaced PW	Spaced PW	Unspaced W	Spaced W	Unspaced PW	Spaced PW
MN	Female	59	8	Left	3	13*	11*	7*	0	0	2*	5*	0	1*	4*	3°
AL	Male	69	8	Right	2	0	0	1	0	0	3*	0	1*	2*	15*	3*
PF	Female	82	9	Right	2	2	4*	4*	0	4*	3*	5*	4*	1*	9*	5°

W, words; PW, pseudowords; L, left; R, right. * pathological performance; ° borderline performance.

### Patient MN

Patient MN, a 59-year-old woman with 8 years of education, was evaluated three months after a right-sided ischemic cerebrovascular event involving fronto-temporal-parietal areas. She exhibited moderate-to-severe left SN in both the Bell and Apple cancellation tests (see Table 6), she omitted the most left-sided element in reading one out of six sentences, and she exhibited moderate personal neglect (4/9 score). In pseudoword reading, the number of omissions increased with letter spacing (two errors in the unspaced condition vs. five errors in the spaced condition). Conversely, substitutions remained grossly stable with spacing (four errors in the unspaced vs. three errors in the spaced condition). In addition, most errors occurred on the left side of the stimuli. Examples of omissions made by the patient include “pesideo”—sideo, “sefio”—efio, and “bartose”—artose; examples of substitutions include “maverta”—naverta, “mefre”—nefre, and “tulcalo”—vulcalo. The patient’s performance is consistent with ND.

### Patient AL

Patient AL, a 69-year-old man with 8 years of education, underwent a neuropsychological assessment two months after an ischemic stroke involving the cortico-subcortical region of the right posterior frontal lobe, extending partially into the deep structures of the corona radiata on that side and to the premotor cortex. No signs of left SN were detected on cancellation tasks (Table 6). In pseudoword reading, the patient exhibited a relatively low number of omissions (three errors for unspaced pseudowords vs. no errors for spaced pseudowords), while a notable decrease in the number of substitutions in the spaced (three errors) compared to the unspaced condition (15 errors) was observed. Furthermore, a qualitative analysis of error classification highlighted that omission errors were not localized in the left portion of the stimulus and were always associated with substitution errors (“rofando”—rofago, “vimpo”—vibo, and “dustore”—tudore). This performance is consistent with CD.

### Patient PF

Patient PF is an 82-year-old woman with 9 years of education. She underwent a neuropsychological assessment three months after an ischemic stroke involving the right temporo-parietal areas. She exhibited signs of left SN, in both the egocentric and allocentric reference frames on the Apple cancellation test (Table 6). She obtained pathological performance on the line (left–right omission difference: 9), star (left-right omission difference: 13), and letter (left-right omission difference: 21) cancellation tests, and in the line bisection task (standardized % score: +52.67). She omitted an element during the complex figure copy, and she committed errors in reading six out of six sentences, without exhibiting signs of personal neglect. In pseudoword reading, the number of omissions increased with letter spacing (three errors in the unspaced condition, nine errors in the spaced condition). Conversely, substitutions decreased with spacing (thirteen errors in the unspaced condition, six errors in the spaced condition). This case is consistent with the presence of both ND and CD.

## Discussion

This study describes a novel reading test to evaluate acquired peripheral dyslexias. Two versions of the test are provided (list A and B), each consisting of 72 stimuli, divided into four sub-lists: unspaced words, unspaced pseudowords, spaced words, and spaced pseudowords. Errors are classified according to two criteria: word-based and letter-based (WBC and LBC, respectively), the latter being more informative of acquired peripheral dyslexia (see Daini et al., 2025). From a sample of neurologically unimpaired individuals, stratified by age, sex, and education level, regression-based norms and cutoff scores for the number of errors are established for both pseudoword lists, according to the WBC and LBC (divided into omissions, substitutions, and additions). No errors were observed in word reading, both unspaced and spaced, by the normative sample.

Reading tests should be included in a sensitive combination of tools not only to assess SN after stroke (Azouvi et al., 2023), but also in right brain-damaged patients, in whom it may help detect other types of acquired peripheral dyslexia (e.g., CD). However, for the Italian population, very few formal tests are available to investigate the presence of ND, mostly requiring the reading of sentences, such as the sentence reading test (Zoccolotti et al., 1989), or text, as the subtests of reading a menu and an article included in the Behavioral Inattention Test (Wilson et al., 1987).

Concerning more specifically single-word reading, only experimental tasks have been published without proper norms. One example is the list of words and legal and illegal nonwords used by Vallar et al. (1996) to detect the presence of ND and to evaluate whether reading performance is influenced by lexicality and by the phonological status of the stimulus, with counting of errors being word-based. However, the legal nonword stimuli used could promote lexicalization errors, as they differed from real words only at the left end (e.g., ipologio–orologio “clock”), whereas the list of illegal nonwords may be too difficult for patients.

The SWAP reading test, presented here, accompanied by norms from a large neurologically unimpaired sample, allows a more fine-grained interpretation of the reading performance of right-brain-damaged patients. It permits the assessment of lexical effects, such as the facilitation observed for real words *versus* pseudowords, as well as an attentional, visual-perceptual mechanism, due to the presence of spacing. Indeed, right-brain-damaged patients with CD typically show a reduction of less-lateralized substitution errors with spaced stimuli (Daini et al., 2021, 2025). On the other hand, lateralized omissions have been shown to increase with spacing, being related to the same mechanism as SN (Daini et al., 2013, 2025; Martelli et al., 2011; Weinzierl et al., 2012) and co-occurring with altered eye movements (Daini et al., 2013; Primativo et al., 2013, 2015).

Our findings show that certain demographic features, such as age and education, are significant predictors of reading performance. Pegoraro et al. (2024) reported similar results in the older adult population. Specifically, they found an age-related decline in reading performance mediated by the score on the symbol digit modalities test (SDMT, Smith, 1982), which assesses processing speed, sustained and selective attention, and working memory. The authors suggested that the SDMT, as well as reading, involves complex functions that are more likely to be affected by aging. Concerning education, its role in predicting word and pseudoword reading performance can be explained in two ways: (i) higher levels of education are associated with better reading skills (e.g., McCarron and Kuperman, 2022); (ii) education is considered a proxy for cognitive reserve (Stern, 2012), and a substantial body of scientific literature suggests that higher cognitive reserve is associated with a slower decline in several cognitive functions during healthy and pathological aging (e.g., Panico et al., 2023; Veronelli et al., 2025b).

When comparing the two lists, we found a positive relationship between spaced stimuli (i.e., pseudowords), while between unspaced stimuli, the correlation was not significant. This result could be explained by the low number of errors made by the enrolled participants, despite the fact that the stimuli were carefully balanced according to numerous psycholinguistic variables across lists. Although a relationship for the unspaced stimuli between the two lists emerged, it is not strong enough to suggest using them as parallel versions of the same test.

The present findings in neurologically healthy participants are consistent with the well-known lexical effect (favoring words) shown by ND (see Arduino et al., 2002) and CD patients (Daini et al., 2025). Indeed, as typically found in reading tasks, no errors were made with real words in both unspaced and spaced conditions. Errors made by healthy individuals in reading pseudowords may indicate that unfamiliar stimuli, which do not have a semantic counterpart or a representation in memory, did not undergo this facilitation, relying more heavily on phonological processing (see McNorgan et al., 2015, for a meta-analytic neuroimaging review).

Furthermore, in enlarging the space between letters in pseudowords, neurologically healthy participants made more substitutions and additions. This result differs from what we found in right-brain-damaged patients with CD, for whom spacing decreases the number of substitutions in pseudowords (e.g., Daini et al., 2025). Here, an increase in the number of errors in reading spaced compared to unspaced pseudowords was found for substitutions and additions, and not for omissions, suggesting different underlying processing mechanisms for omissions and substitutions also in neurologically healthy participants.

Concerning mechanisms, according to the study by Daini et al. (2021), substitution errors would be due to a deficit in the control of the focal component of selective attention. Three patients in the first experiment and four patients in the second experiment showed a reading deficit characterized by a prevalence of substitution errors, which decreased with the increase in spacing between letters (i.e., crowding). These patients also showed a lack of facilitation of an optimal cue of focal attention in a simple detection task.

On the other hand, in normal conditions (i.e., without a visual-perceptual deficit and CD), progressively increased spacing between letters may alter the stored visual word form, interfering with the process of visual recognition of words (Cohen et al., 2008) and reading. The present results suggest that in neurologically unimpaired participants, increased spacing could interfere with visual processing of unfamiliar letter strings and phonological decoding. Conversely, when the reading process is impaired by visual-perceptual and focal attention disorders, such as crowding, spacing facilitates reading, reducing the number of substitutions.

The SWAP reading test was specifically created to identify the spacing effect in peripheral dyslexia, which allows CD to be distinguished from ND in patients with right-hemisphere focal lesions (Martelli et al., 2011; Daini et al., 2013, 2021, 2025). The tool, however, having lexicality (words and pseudowords) among the variables considered, can also be useful for detecting an acquired central type of dyslexia such as phonological dyslexia, in which pseudowords are read with difficulty, and word reading remains grossly uncompromised. Since our test does not manipulate the presence of regularly and irregularly stressed words, which for the Italian language permits disentangling between lexical and nonlexical reading, it does not allow the identification of surface dyslexia.

Moreover, peripheral dyslexias, although rarely, can also be seen in some patients with neurodegenerative disease, in particular posterior cortical atrophy (PCA). Crutch and Warrington (2007) were the first to describe visual crowding in individuals with PCA in reading tasks and with spacing manipulation. They suggested that the reading deficits of PCA patients were not only due to more general visual deficits but also to an additional crowding deficit due to excessive integration of letter features (Crutch and Warrington, 2009).

Subsequently, Yong et al. (2014) compared PCA and typical Alzheimer’s Disease (tAD) patients in word reading tasks with spacing manipulation as an experimental variable. They found that reading performance in patients with PCA was significantly less accurate and slower than that of both tAD patients and controls, and sensitive to letter spacing. These studies suggest that the SWAP test may prove useful in the assessment of patients with PCA, although this possibility requires empirical validation. Future studies should also investigate its usefulness in the assessment of MCI patients at a preclinical stage.

## Conclusion

In conclusion, currently available Italian word-reading assessment tools, based on psycholinguistic variables, are mainly dedicated to central dyslexias. An accurate assessment of peripheral dyslexias, with a focus on visuo-perceptual variables that may modulate performance, is still lacking. The availability of regression-based normative data of the Italian population will allow the clinical use of the SWAP reading test. Clinical examples demonstrate the test’s usefulness in distinguishing between ND and CD and the different mechanisms underlying these conditions.

## Data Availability

Publicly available datasets were analyzed in this study. This data can be found here: https://osf.io/vjs7y/?view_only=7430f800707c449c977bbd30110732da.
