# Supplementary material for: The spacing words and pseudowords reading test: normative data to measure peripheral acquired dyslexia in the Italian adult population
Source: Front Psychol. 2026 Sep 3;17:1911950. doi: 10.3389/fpsyg.2026.1911950 (PMC13581806; doi:10.3389/fpsyg.2026.1911950)
Supplement: Supplementary file 1 [file Supplementary_file_1.docx]

**Supplementary Materials**

Stimuli included in list A and B.

**List A**

| A-WORDS | A-PSEUDOWORDS | A-SPACED WORDS | A-SPACED PSEUDOWORDS |
| --- | --- | --- | --- |
| testa | rofando | bastone | astimpo |
| scatola | prafe | furto | notra |
| taverna | pesideo | istinto | efela |
| libro | vimpo | preda | truco |
| miele | nerta | vescovo | riepe |
| fango | maverta | vizio | ginfolo |
| merce | dumio | canzone | puoga |
| piscina | tescofo | frase | benefra |
| sedia | lirto | tenebra | bartose |
| luogo | cifiere | diluvio | puoro |
| pistola | dustore | edera | pintoma |
| cielo | mieco | festa | tesba |
| residuo | niscifa | cornice | sefio |
| giudice | mefre | stomaco | dalevio |
| vulcano | tornise | carcere | misfelo |
| simbolo | giafide | madre | batro |
| mistero | folgo | scudo | tulcalo |
| suora | ciase | cuoio | fantoge |

**List B**

| B-WORDS | B-PSEUDOWORDS | B-SPACED WORDS | B-SPACED PSEUDOWORDS |
| --- | --- | --- | --- |
| argento | calisia | mondo | coltere |
| fascino | mirento | pugno | puodo |
| pulpito | stibe | candela | etefa |
| catrame | vulfito | pentola | siafa |
| polvere | fiegolo | colpa | saniepo |
| fuoco | barno | pigiama | depotio |
| vicenda | tusmino | scienza | trisa |
| fiume | faula | bugia | vamba |
| barba | vonse | turno | Pugiata |
| nazione | colfi | lampada | Pertova |
| palma | crinido | spada | manione |
| prosa | lanzata | cuscino | Dugna |
| diavolo | mastepa | paura | Puaso |
| guaio | bomia | ritmo | tembaro |
| valigia | bindo | vetro | bicesda |
| cuore | sefro | brivido | Acina |
| erede | pafento | tamburo | fatrane |
| ponte | sinfa | guancia | galme |

Mean values (SD) of the psycholinguistic variables regarding words in the two lists (A and B), in the two conditions (unspaced and spaced).

|  | **List A-**  **Unspaced Words** | **List B-**  **Unspaced Words** | **List A-**  **Spaced Words** | **List B-**  **Spaced Words** |  |
| --- | --- | --- | --- | --- | --- |
| **AoA** | 3.49 | 3.55 | 3.71 | 3.20 |  |
| **FAM** | 6.27 | 6.14 | 6.22 | 6.19 |  |
| **IMM** | 5.20 | 4.72 | 4.82 | 4.88 |  |
| **CONC** | 5.76 | 5.22 | 5.13 | 5.03 |  |
| **FQ** | 119.00 | 117.00 | 120.00 | 124.00 |  |
| **FQ-BIGR** | 10.87 | 10.82 | 10.85 | 10.70 |  |
| **SYLL** | 2.50 | 2.56 | 2.56 | 2.50 |  |
| **LETTERS** | 6.00 | 6.00 | 6.00 | 6.00 |  |
| **N-SIZE** | 1.00 | 0.78 | 1.00 | 0.89 |  |
|  | **List A-Unspaced Words** | **List A-Spaced Words** | **List B-Unspaced Words** | **List B-Spaced Words** |  |
| **AoA** | 3.49 | 3.71 | 3.55 | 3.20 |  |
| **FAM** | 6.27 | 6.22 | 6.14 | 6.19 |  |
| **IMM** | 5.20 | 4.82 | 4.72 | 4.88 |  |
| **CONC** | 5.76 | 5.13 | 5.22 | 5.03 |  |
| **FQ** | 119.00 | 120.00 | 117.00 | 124.00 |  |
| **FQ-BIGR** | 10.87 | 10.85 | 10.82 | 10.70 |  |
| **SYLL** | 2.50 | 2.56 | 2.56 | 2.50 |  |
| **LETTERS** | 6.00 | 6.00 | 6.00 | 6.00 |  |
| **N-SIZE** | 1.00 | 1.00 | 0.78 | 0.89 |  |
| AoA: Age of Acquisition; FAM: familiarity; IMM: imaginability; CONC: concreteness; FQ: word written frequency; FQ-BIGRAM: bigram frequency; SYLL: number of syllables; LETTERS: number of letters; N-SIZE: orthographic neighborhood size. No significant differences between stimuli sets were detected at the Kruskall-Wallis test (p> .2, for all comparisons). | | | | | |

Mean values of the psycholinguistic variables regarding pseudowords in the two lists (A and B), in the two conditions (unspaced and spaced) and two tailed t-test comparison between and within list conditions.

|  | **List A- Unspaced Pseudowords** | **List B- Unspaced Pseudowords** | **List A- Spaced Pseudowords** | **List B- Spaced Pseudowords** |  |
| --- | --- | --- | --- | --- | --- |
| **LETTERS** | 6 | 6 | 6 | 6 |  |
| **SYLL** | 2.6 | 2.6 | 2.6 | 2.6 |  |
| **N-SIZE** | 0.28 | 0.28 | 0.22 | 0.28 |  |
| **FQ-BIGR** | 11.51 | 11.46 | 11.45 | 11.53 |  |
|  |  |  |  |  |  |
|  | **List A- Unspaced Pseudowords** | **List A- Spaced Pseudowords** | **List B- Unspaced Pseudowords** | **List B- Spaced Pseudowords** |  |
| **LETTERS** | 6 | 6 | 6 | 6 |  |
| **SYLL** | 2.6 | 2.6 | 2.6 | 2.6 |  |
| **N-SIZE** | 0.28 | 0.22 | 0.28 | 0.28 |  |
| **FQ-BIGR** | 11.51 | 11.45 | 11.46 | 11.53 |  |
| LETTERS: number of letters; SYLL: number of syllables; N-SIZE: orthographic neighborhood size; FQ-BIGRAM: bigram frequency. No significant differences between stimuli sets were detected at the t-test and Kruskall-Wallis test (p> .8, for all comparisons). | | | | | |
